# Gene Expression Dosage Regulation in an Allopolyploid Fish

**DOI:** 10.1371/journal.pone.0116309

**Published:** 2015-03-19

**Authors:** I Matos, M. P. Machado, M. Schartl, M. M. Coelho

**Affiliations:** 1 CE3C—Centre for Ecology, Evolution and Environmental Changes, Faculdade de Ciências, Universidade de Lisboa, 1749–016 Lisboa, Portugal; 2 University of Würzburg, Biozentrum, Physiological Chemistry, Am Hubland, Würzburg, Germany; 3 Comprehensive Cancer Center, University Clinic Würzburg, Josef Schneider Straße 6, 97074 Würzburg, Germany; Niels Bohr Institute, DENMARK

## Abstract

How allopolyploids are able not only to cope but profit from their condition is a question that remains elusive, but is of great importance within the context of successful allopolyploid evolution. One outstanding example of successful allopolyploidy is the endemic Iberian cyprinid *Squalius alburnoides*. Previously, based on the evaluation of a few genes, it was reported that the transcription levels between diploid and triploid *S*. *alburnoides* were similar. If this phenomenon occurs on a full genomic scale, a wide functional ‘‘diploidization’’ could be related to the success of these polyploids. We generated RNA-seq data from whole juvenile fish and from adult livers, to perform the first comparative quantitative transcriptomic analysis between diploid and triploid individuals of a vertebrate allopolyploid. Together with an assay to estimate relative expression per cell, it was possible to infer the relative sizes of transcriptomes. This showed that diploid and triploid *S*. *alburnoides* hybrids have similar liver transcriptome sizes. This in turn made it valid to directly compare the *S*. *alburnoides* RNA-seq transcript data sets and obtain a profile of dosage responses across the *S*. *alburnoides* transcriptome. We found that 64% of transcripts in juveniles’ samples and 44% in liver samples differed less than twofold between diploid and triploid hybrids (similar expression). Yet, respectively 29% and 15% of transcripts presented accurate dosage compensation (PAA/PA expression ratio of 1 instead of 1.5). Therefore, an exact functional diploidization of the triploid genome does not occur, but a significant down regulation of gene expression in triploids was observed. However, for those genes with similar expression levels between diploids and triploids, expression is not globally strictly proportional to gene dosage nor is it set to a perfect diploid level. This quantitative expression flexibility may be a strong contributor to overcome the genomic shock, and be an immediate evolutionary advantage of allopolyploids.

## Introduction

In polyploid lineages resulting from hybridization (allopolyploids), the combination of homeologous chromosomes from divergent species promotes a multitude of biological events [1]. Heterozygosity, divergence of duplicate genes, and novel gene interactions lead to genetic and phenotypic variability [2] that are stably and successfully maintained in these lineages [1]. Allopolyploids are, in this scope, great evolutionary projects full of opportunities for selection and adaptation. On the other hand, allopolyploid lineages have to face an important challenge, namely to overcome genomic shock caused by the simultaneous high level of heterozygosity (due to hybridization) and gene dosage increase (due to polyploidy) [3]. However, mostly plants and invertebrates but also lower vertebrates, deal with these challenges very successfully [4] as they survive and perpetuate. The evolutionary success of several animal allopolyploid lineages like *Squalius alburnoides* [5], *Rana esculenta* [6], *Bufo viridis* [7] or *Poecilia formosa* [8], outdates research that suggests that the fate of (allo)polyploids is a rapid extinction, and suggests that such animals might developed mechanisms that stabilize their genomes as already widely reported in plants [9].

In allopolyploid plants, the reduction of gene redundancy towards a functional diploidization (dosage compensation) has been pointed out as a way to cope with gene dosage increase [10], but in vertebrates this hypothesis has been scarcely investigated. However, the recent recognition that hybridization and polyploidy are much more frequent in animals than previously inferred [11] and that this might have significantly shaped vertebrate genomes [4] highlighted the importance to extend these studies further than to allopolyploid plants and invertebrates. In a first attempt to study gene expression regulation in a vertebrate allopolyploid context, the expression level of 7 genes (gene set encompassing tissue specific and housekeeping genes), were evaluated and the occurrence of a compensation mechanism was reported in the allopolyploid cyprinid *Squalius alburnoides* [12]. In this fish, for those first analysed genes, the existence of a dosage compensation mechanism that brings transcript levels in triploids to the diploid state was shown. Yet, the genomic extension of the phenomenon remains unknown. It may be a global gene dosage compensation event, acting without exception throughout the whole genome. On the other hand, a certain number of genes may escape the dosage-regulation mechanism, or dosage compensation may be restricted to specific subsets of genes, or any other still unformulated conjecture [12]. However, either taking place on a full genomic scale or only partially, the occurrence of similar transcription levels between diploids and triploids can be a relevant factor contributing to the success and perpetuation of polyploids among lower vertebrates. Analyses of entire transcriptomes (RNA-seq), available in the meantime, are now the imminent choices to disentangle this kind of questions, [13, 14, 15]. RNA-seq allows in a fast and cost-effective way to do a simultaneous qualitative and quantitative analysis of complex transcriptomes [16]. It showed to be a general improvement compared to microarrays [17] and was extensively validated by qPCR, exceeding it in range [14, 18].

**Fig 1:**
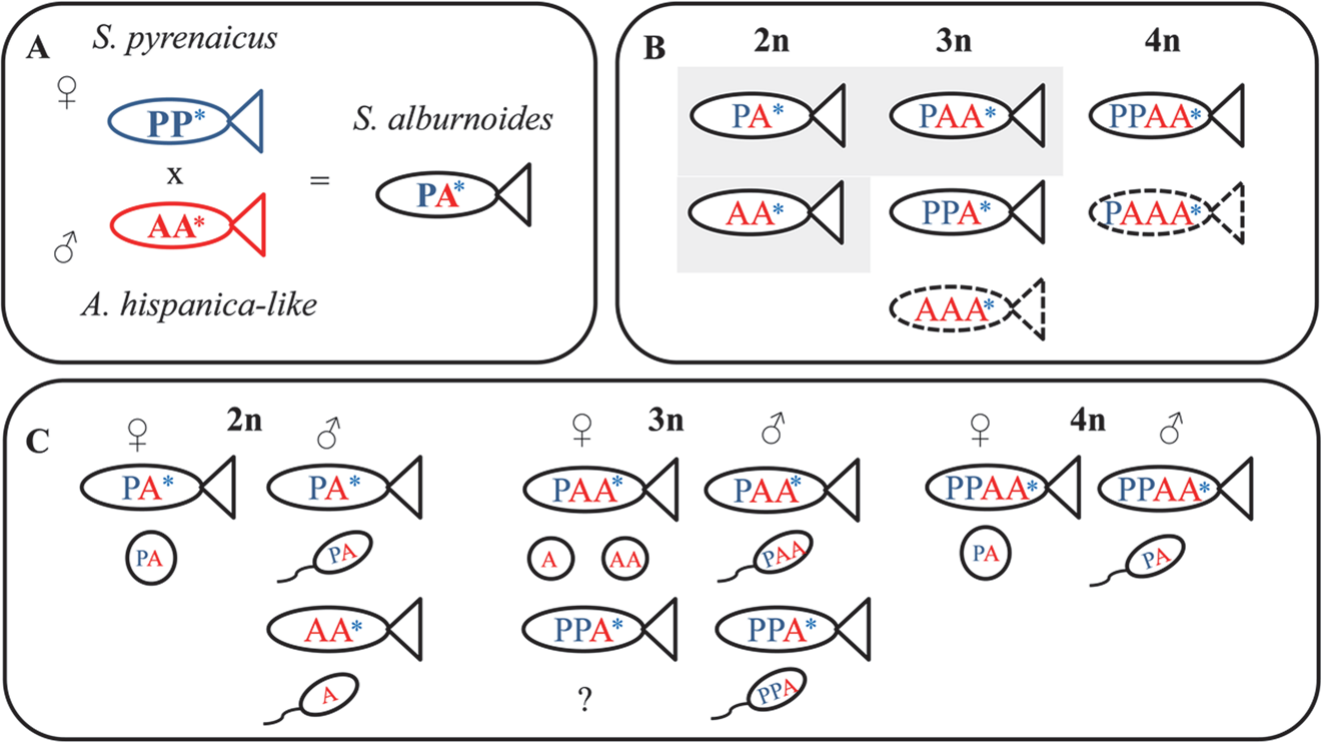
*S*. *alburnoides* complex simplified overview. A) Initial hybridization event in the origin of the complex. B) Diversity of genomotypes found in the main southern portuguese river basins. In gray background are the *S*. *alburnoides* genomotypes more frequent in nature, which are in focus in this work. Dashed lines indicate naturally occurring but rare genomotypes. C) Diversity of gametes, produced through a variety of mechanisms—clonally, by hybridogenesis, meiotic hybridogenesis or normal meiosis, depending on the sex and genomic composition of the individual. Asterisk represents mitochondrial genotype: blue from *S*. *pyrenaicus* and red from *A*. *hispanica*-like.

*Squalius alburnoides* is an allopolyploid cyprinid, resulting from interspecific hybridization between females of *Squalius pyrenaicus* (P genome) and males of a now extinct species related to *Anaecypris hispanica* (A genome) [5, 19] (Fig. 1A). *S*. *alburnoides* natural populations are composed of animals of different ploidy levels and genomic constitutions (Fig. 1B) referred to as genomotypes, and include fertile sexual and non-sexual forms (Fig. 1C) Currently, in the Iberian southern basins the predominant *S*. *alburnoides* genomotypes are the hybrid triploid PAA, diploid PA, and the parental-like diploid AA. Individuals of AA genomotype are all males, and are only reconstituted from hybrids within the complex [5], so despite the homogenomic *A*. *hispanica*-like nucleus, they carry P mitochondrial DNA and are called nuclear non-hybrids (Fig. 1C).

In this work, our goal is to expose how an evolutionary successful allopolyploid vertebrate, the cyprinid *S*. *alburnoides*, deals on the transcriptional level with the genomic stress derived from hybridization and polyploidy. Also, this study aims to contribute to understand the role of gene dosage compensation in the *S*. *alburnoides* breeding complex.

In the present work, the quantitative expression profiles of diploid and triploid *S*. *alburnoides* were compared after RNA-sequencing and *de novo* assembly of the *S*. *alburnoides* transcriptome. Since it is known that there are many transcriptional changes from juveniles to adults [20] and that gene expression patterns can be tissue-specific [12, 21], RNA-seq was done both from whole bodies and at a single tissue level.

However, the RNA-seq transcript profiling experiments the differences in expression of a gene between two samples are in fact differences in expression per unit of RNA or “per transcriptome. To directly infer global expression dosage responses from the RNA-Seq transcript profiling experiments the transcriptomes compared must be of equal size. Without information about the sizes of the transcriptomes compared, direct assumptions about the expression per gene copy or expression per cell drawn from the transcriptome-normalized expression can be flawed [22; 23]. Based only on the expression per transcriptome, the differences in expression per cell that are proportional to the change in the total transcriptome size will appear as equal expression per transcriptome [23]. Consequently, we also estimated the relative transcriptome size from liver samples between diploid and triploid *S*. *alburnoides* hybrids.

## Materials and Methods

### Fish samples and genotyping

#### Adult specimens

From the area of sympatry of *S*. *pyrenaicus* and *S*. *alburnoides*, in southern Portuguese river basins, a total 20 specimens (6 *S*. *pyrenaicus* and 14 *S*. *alburnoides*) were collected to perform experimental crosses and provide adult biological material. Individuals were collected from tree locations: Algarve basin, Almargem stream (29 S; 622495.24 m E; 4113964.49 m N (UTM)); Guadiana basin, Oeiras stream (29 S; 604985.29 m E; 4164883.94 m N (UTM)) and Tejo basin, Cobrão stream (29 S; 606212.42 m E; 4398531.98 m N (UTM)). Sampling locations were chosen according with the legal permits and considering the existence of a differential geographical distribution of genomotypes, different relative frequencies of each genomotype in each river basin and the fact that diploid and triploid *S*. *alburnoides* are not morphologically distinguishable [5]. All specimens were adults, sexually mature (determined by obvious abdominal distension and gametes releasing upon slight abdominal pressure) and approximately one to three years old (estimated from the length of each specimen). Fish were captured by electrofishing and brought alive to the laboratory. Each fish was photographed to posterior identification by Scaleprinting [24]. Also, DNA was obtained from fin clips and the specimens were genotyped according to [25]. Fish were acclimated for three weeks in high-quality glass tanks (30 l capacity) equipped with filtration units, under the same standard conditions of light (14 hours light, 10 hours dark), temperature (22°C ±1 °C), water quality (pH between 6.5 and 7.30) and nutrition (twice a day feeding with frozen anthemia and commercial fish food flacks).

Fourteen individuals (6 PA; 6 PAA; 1 AA and 1 PP) were sacrificed and organs were dissected and preserved in RNA later (Ambion) at -20°C.

#### Juveniles

During the reproductive season, and before its use in the previous section, all adults of *S*. *alburnoides* and *S*. *pyrenaicus* (previously genotyped) and visibly sexually maturated were used to perform defined experimental crosses in order to obtain progenies specifically with PAA, PA, AA and PP genomotypes [5, 26]. For each cross, eggs and sperm were collected (by gentle abdominal pressure) from the selected individuals and used for embryo production in petri dishes. Successful fertilization was assessed by observation under the stereoscope after 2h. Viable progeny wit PP genomotype was not obtained. At least 2 viable progenies of each, putatively of AA, PA and PAA genomotypes were reared in high-quality glass tanks (5 l capacity) equipped aerating units, under the same standard conditions of light (14 hours light, 10 hours dark), temperature (20°C ±1°C), water quality (pH between 6.5 and 7.30) and nutrition (twice a day feeding with commercial powder food for fish larvae). 30 days after hatching (dah) several siblings from the same clutch and from each genotype were collected and preserved in RNA later (Ambion) at-20°C. Simultaneous extractions of DNA and total RNA were performed with the AllPrep DNA/RNA Mini Kit (Qiagen). The extracted DNA was used to assess the genomic composition of the selected progenies according to [25].

### Library construction and sequencing

Total RNA extracted with the AllPrep DNA/RNA Mini Kit (Qiagen) was DNase treated on-column with the RNase-free DNase Set (Qiagen). At least 15 μg of RNA were obtained per sample. Integrity evaluation and quantification of the extracted RNA was performed with Nanodrop 1000 (Thermo Scientific) and 2100 Bioanalyser (Agilent Technologies). All samples presented a RIN higher than 8.5 (Bioanalyser). Normalized juvenile cDNA libraries were homemade prepared according to [27]. Non-normalized liver libraries were prepared with TruSeq RNA Sample Preparation Kit (Illumina) according to the Illumina specifications. All libraries were paired-end sequenced using Illumina HiSeq 2000.

#### Juvenile samples

Three barcoded RNA libraries were constructed: juvenile-AA, juvenile-PA and juvenile-PAA. For the construction of the 3 libraries RNA was purified from whole bodies of pools of 4 larvae of each genomotype at 30 dah. At this age, all major organs (except the reproductive systems that are not yet fully defined) are already formed in all 3 investigated genomic forms of *S*. *alburnoides* (unpublished data). We did pooling of individuals in order to obtain the minimal amount of RNA required for library construction and sequencing. For each library only siblings from the same cross were used. The 3 libraries were sequenced producing 12 Gb clean data (~ 4Gb per library) in 3 data sets (juv-AA; juv-PA; and juv-PAA) of Illumina HiSeq short paired-end sequence reads (90 bp). The output statistics of sequenced data is available as S1 Table.

#### Liver samples

Four barcoded RNA libraries were constructed: one for *S*. *pyrenaicus* (liver- PP) and three for *S*. *alburnoides* (liver-AA, liver-PA and liver-PAA). For the construction of the libraries RNA was purified from livers, independently for each sample/library. The 4 libraries were sequenced producing 4Gb of clean data (~1Gb per library) in 4 data sets (liv-AA; liv-PA; liv-PP and liv-PAA) (short paired-end sequence reads around 50 bp). The output statistics of sequenced data is available as S2 Table.

### Processing of RNA-seq data for gene expression quantification

The raw data of juv-AA, juv-PA, and juv-PAA were processed into clean data by removing reads with adaptors, reads with more than 5% of unknown nucleotides and reads where more than half of the bases’ quality values were less than 5. Also, orphan reads were excluded.

Transcriptome de novo assembly was carried out with Trinity [28]. Assemblies were taken into further processes of sequence splicing and redundancy removing with the sequence clustering software TGICL [29]. After clustering, UniGenes were divided in two classes: clusters (prefix CL) and singletons (prefix unigene) (Statistics of assembly quality provided as S3 Table). blastx alignment (e-value < 0.00001) between unigenes and protein databases (nr, Swiss-Prot, KEGG, COG) was performed, and the best aligning results were used to decide sequence direction. When results of different databases conflicted, the priority order of nr, Swiss-Prot, KEGG and COG was followed (statistics of annotation results provided as S4 Table). Unigenes that were not aligned to any of these databases were scanned by ESTScan (v2.1) [30], to decide the sequence direction. The expression level of each unigene was calculated as FPKM, defined as fragments per kilobase of exon model per million mapped fragments [31], with the Cufflinks package (v0.9.3).

Concerning raw sequencing data of liv-AA, liv-PA, liv-PP and liv-PAA, quality filtering was performed: low quality (phred score<20), and ambiguous nucleotides were trimmed off and the quality assessed using FastQC v0.10.1. Reads were mapped to the *Danio rerio* reference genome (Ensembl *Danio rerio* genome Zv9.69) using Stampy v1.0.21 (substitution rate of 11% and no multiple hits allowed) (Mapping statistics are presented as S2 Table). To use the mapping approach the divergence rate between *D*. *rerio* and *S*. *pyrenaicus* (11%) and *D*. *rerio* and *S*. *alburnoides* AA genomotype (10%) had to be assessed and the higher value was used (11%). Fragments mapped into genes were counted using HTSeq v0.5.3p9 (htseq-count option for no stranded data). FPKM values were calculated using fragment counts from HTSeq and total fragments mapped obtained with Samtools v0.1.18. (flagstat option, counting pair reads plus singletons mapped). Differentially expressed genes were calculated using Bioconductor edgeR package v3.0.8 (for a FDR<0.05) after data normalization using Bioconductor EDASeq package v1.4.0 (first normalized to gene length and second to the libraries size).

### Comparative analysis of expression levels

Expression differences were obtained by dividing the normalized expression values (FPKM) in one library by the corresponding expression value of the same transcript in each other library (fold change) independently for juveniles and livers. The quantitative comparative profiles were displayed through orderly plotting of log_2_ (fold change). The value of |log_2_(Ratio)|<1 was considered to be the threshold for similar expression. A false discovery rate (FDR) ≤ 0.05 was used as cutoff threshold to determine the significance of differential expression (FDR correction, version for dependent tests, applied to the raw p-value of all transcripts).

Also, the observed gene expression level of each transcript in the hybrids was compared to an expected expression level if P and A alleles are expressed exactly at the same level as in the non-hybrid situation (additivity expectation). The expression level of each gene in the parental diploids (AA and PP) was used to calculate the expected additive expression for each gene in both hybrids. Then, the observed expression value (obs) of each transcript was divided by its corresponding expected additive value (exp), both in liv-PAA and in liv-PA. These ratios were log_2_ transformed and when within the interval -1<log_2_(observed FPKM/expected FPKM)<1, the transcripts were considered as additively expressed.

When appropriate, the χ-square test was applied to the data and is indicated.

### Functional analysis

Functional enrichment analyses were carried out using DAVID Bioinformatics Resource 6.7 (http://david.abcc.ncifcrf.gov/). The top blastx hits in nr database corresponding to each *S*. *alburnoides* unigene were used as customized reference background for juveniles’ data set. As liver gene expression reference background we used the mapped genes with expression in at least one of the 4 liver libraries. DAVID sorting thresholds were changed to EASE score (modified Fisher exact test) ≤ 0.01. Significant enrichment was only considered when Benjamini corrected p-value ≤ 0.05.

### Data accessibility

Files containing the clean reads for *S*. *alburnoides* juvenile transcriptome assembly and the clean reads for the *S*. *alburnoides* liver transcriptome mapping are available in ArrayExpress, accession number E-MTAB-3174.

### qRT-PCR genome-normalized expression assay and relative transcriptome size estimation

RNA and gDNA (total nucleic acid [TNA]) were co-extracted from RNA later (Ambion) preserved livers according to the extraction protocol described in [32] with minor modifications. TNA were extracted independently from 5 livers of each diploid and triploid hybrid (PA and PAA; total n = 10) and the presence of both RNA and gDNA confirmed in a 1,5% agarose gel for all 10 samples. 1 μg of TNA per sample was reversed transcribed with RevertAid First Strand cDNA Synthesis Kit (Fermentas) with oligo dT primers.

Primers for target genes were designed to be specific to either cDNA or gDNA (S5 Table). For cDNA-specific primers, one or both primers in a pair were designed to span exon-exon splice junctions and for gDNA-specific primers, one or both primers were designed to prime at least partially within an intron. Template specificity was confirmed for all primer pairs by qPCR with cDNA and gDNA templates. Primers specific to cDNA were designed for 6 genes (*rpl8*, *rpl35*, *actb2*, *pabpc1a*, *eef1a* and *rpsa*) and primers specific to gDNA were designed for 3 genes (*rpl8*, *eef1a* and *actb2*) (S5 Table).

The cDNA/gDNA mix was diluted 1:1 in nuclease free-water and was 1 μl used as template for each qRT-PCR reaction.

Real-time PCR reactions were performed in BioRad’s CFX96 Real-time PCR system (C1000 Thermal Cycler). Real-time PCRs were done in a final volume of 10 μl, with SsoAdvanced Universal SyberGreen Supermix (BioRad) in accordance to the specification of the supplier. The thermal cycling protocol was as follows: initial denaturation step at 95°C for 30s, followed by 40 cycles at 95°C for 10s and 60°C for 30s. For each primer pair, we amplified three technical replicates from each of the five biological replicates of each of the two genomotypes. Expression of each target gene (cDNA-specific amplification) was normalized to the geometric mean of amplification from the three gDNA-specific targets. Relative genome-normalized expression values were calculated by the Livak method [33].

We calculated expression per cell in PAA relative to PA as 1,5x the relative expression per genome.

RPKM values from the liver data set, obtained as described above, were taken as the transcript abundance per transcriptome for any given gene.

To estimate the hybrid triploid liver transcriptome relative to the hybrid diploid liver transcriptome the per cell expression ratios from the qRT-PCR assay were divided by the per transcriptome expression ratios from the liver RNA-seq data set.

### Ethics Statement

Fish captures and handling needed the permission of Instituto de Conservação da Natureza e das Florestas (ICNF), the Portuguese national authority and relevant body concerned with protection of wildlife. ICNF considered that our study was not intrusive issuing the permits AFN, fishing credential n° 82/2012 and ICNB license n°142/2012/CAPT. The studied species are not endangered or protected, nevertheless the selected populations for the captures ware not imperiled, and sampling was done avoiding depletion of the natural stock. Electrofishing was performed in low duration pulses to avoid killing juveniles (300 V, 2–4 A) and the transport to the laboratory was made in appropriate aerated containers. The maintenance and use of animals in the animal facility of the Faculdade de Ciências da Universidade de Lisboa had the approval of the Direção-Geral de Veterinária, Direção de Serviços de Saúde e Proteção Animal (DGV-DSSPA), stated in the “ofício circular” n° 99 0420/000/000/9/11/2009. Fish were handled following the recommended ethical guidelines in [34] and at all times, all efforts were made to minimize suffering. All required manipulations for identification, genotyping and to accomplish the experimental crosses were performed under light anesthesia (40 ppm of MS222 dissolved in the water). Fish used for the experimental crosses that were not sacrificed were later returned to their original capture site. The sacrificed individuals were submitted to an overdose of MS222 (400 ppm of MS222 dissolved in the water) and kicky decapitate previously to the organs harvesting to guarantee the death prior to the harvesting.

## Results

### Comparative expression profiling from triploid and diploid juveniles

**Fig 2:**
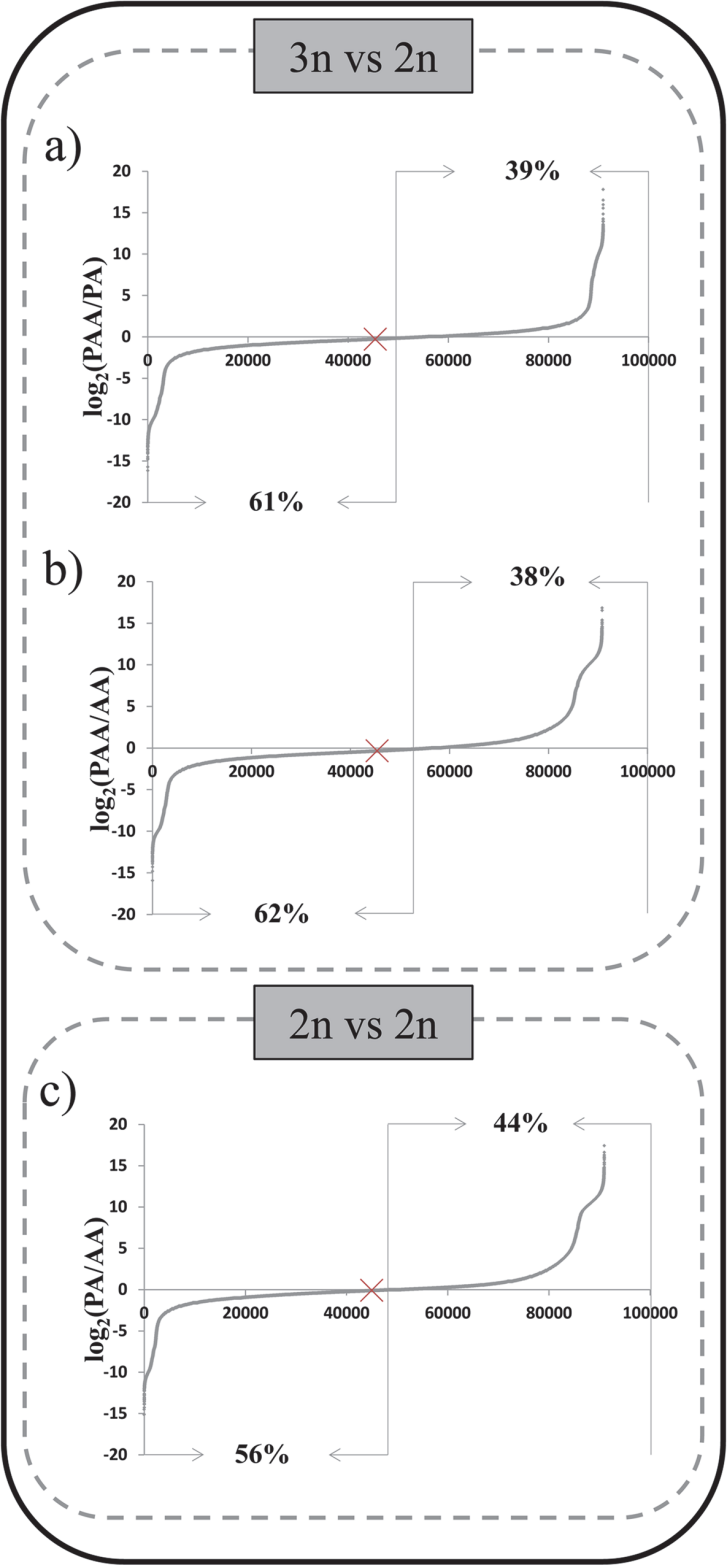
Comparative gene expression profiles between three juvenile genomotypes of the *S*. *alburnoides* complex. Logarithmized ratios of gene expression for each unigene were orderly plotted producing characteristic crescent curves where positive values indicate transcripts with higher expression and negative values transcripts with lower expression. Median is marked with a cross and indicates if most values are positive or negative. For all comparisons, the difference in the number of lower vs higher expressed transcripts is significant (χ-test, p<0.001). The percentages of positive and negative values in each comparison are indicated.

To investigate if the quantitative expression profile of mRNAs changes with ploidy increase we made pairwise comparisons between the expression level profiles of each pair of triploid vs diploid juvenile *S*. *alburnoides* genomotype. We plotted the log_2_ FPKM ratios (juv-PAA/juv-PA) (Fig. 2A) and log_2_ FPKM ratios (juv-PAA/juv-AA) (Fig. 2B), producing crescent curves where positive values indicate mRNAs with higher expression in juv-PAA than in juv-PA or juv-AA, and negative values represent mRNAs with lower expression in juv-PAA then in juv- PA or juv-AA respectively. The same comparative expression profiling was performed between juv-PA/juv-AA (Fig. 2C) to illustrate a comparison at the diploid level.

We observed a significant (χ-test, p<0.001) higher amount of lower expressed unigenes in juv-PAA compared to juv-PA (Fig. 2A) and also (χ-test, p<0.001) in the comparison of juv-PAA with juv-AA (Fig. 2B). This is contrary to what would have been expected from a dosage effect between triploid and diploid organisms. For a dosage effect it would be expected that most transcripts would be higher represented in triploids then in diploids, or similarly represented in case of dosage compensation. Concerning the comparison between juv-PA and juv-AA, where ploidy rise has no part (only hybridization), despite significant (χ-test, p<0.001) a much less conspicuous difference in the number of lower vs higher expressed transcripts is observed (Fig. 2C).

Focusing only on the ploidy level effect, we compared the amount of unigenes that are lower (n = 55545) and higher (n = 35411) expressed between juv-PAA and juv-AA with those lower (n = 50942) and higher (n = 40004) expressed between juv-PA and juv-AA. The expression pattern of the hybrids compared to a diploid non-hybrid is significantly affected (χ-test, p<0.001) by the ploidy level of the hybrids. However, the expression profile of the other parental diploid genomic composition (PP) is needed to make a firm conclusion.

### Comparative expression profiling of livers from diploid and triploid adults

Unlike for juveniles, material from adult *S*. *pyrenaicus* (PP) could be easily obtained. Therefore, a second set of quantitative expression data, using adult tissues (livers) was produced.

**Fig 3:**
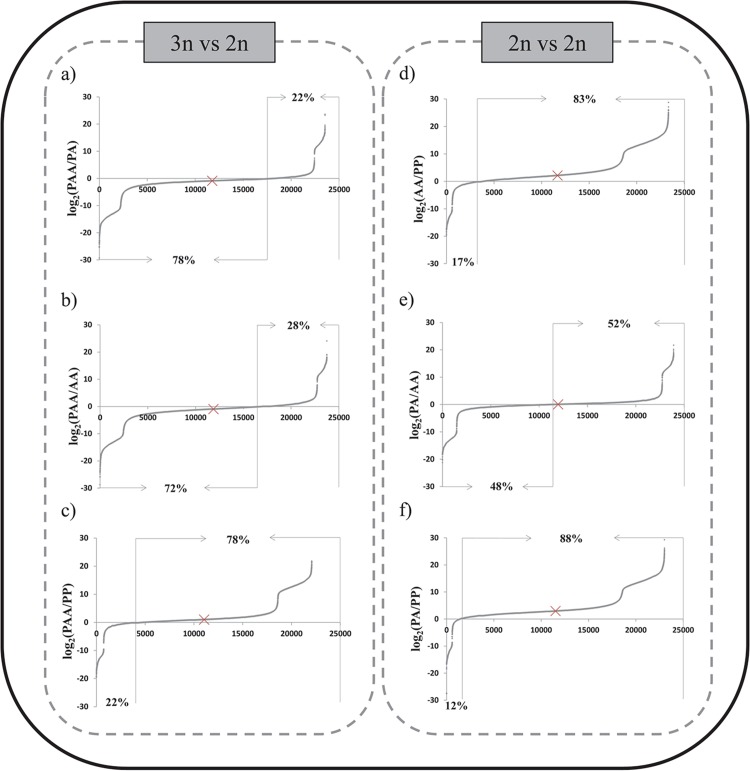
Comparative gene expression profiles in adult liver between the most common forms of the *S*. *alburnoides* complex. Logarithmized ratios of expression for each mapped transcript ware orderly plotted producing a crescent curve where positive values indicate transcripts with higher expression and negative values transcripts with lower expression. Median is marked with a cross and indicates if most values are positive or negative. For all comparisons, the difference in the number of lower vs higher expressed transcripts is significant (χ-test, p<0.001).The percentages of positive and negative values in each comparison are indicated.

As for the juveniles, the expression profiles for the liver were obtained per genomotype, and pairwise comparisons were performed (Fig. 3). The comparison between triploid and diploid levels showed in all cases a significant difference in the number of higher and lower represented transcripts (χ-test, p<0.001) (Fig. 3A-3C), indicating that globally, ploidy level affects the quantitative expression pattern. Despite the higher gene dosage in the triploid, there is a higher amount of lower represented transcripts in liv-PAA compared to both diploid liv-PA and liv-AA (Fig. 3A-3B). This was consistent with what was observed in the whole body juvenile data set (Fig. 2). Moreover, there is a substantially high amount of higher represented transcripts in liv-PAA when compared to liv-PP (Fig. 3C).

The quantitative relative expression patterns within the same ploidy level (2n) were also inspected (Fig. 3D-3F). First, in the comparison between the two parental diploid genomotypes (AA vs PP), it was observed that a massive amount of transcripts were represented at higher levels in AA liver than in PP liver (Fig. 3D). Then, when comparing PA with each one of the parental genomotypes (AA and PP), we observe that in comparison with liv-AA, the difference between lower and higher represented transcripts is only marginal (Fig. 3E), while in the comparison with liv-PP it is really high (Fig. 3F).

### Additivity

**Fig 4:**
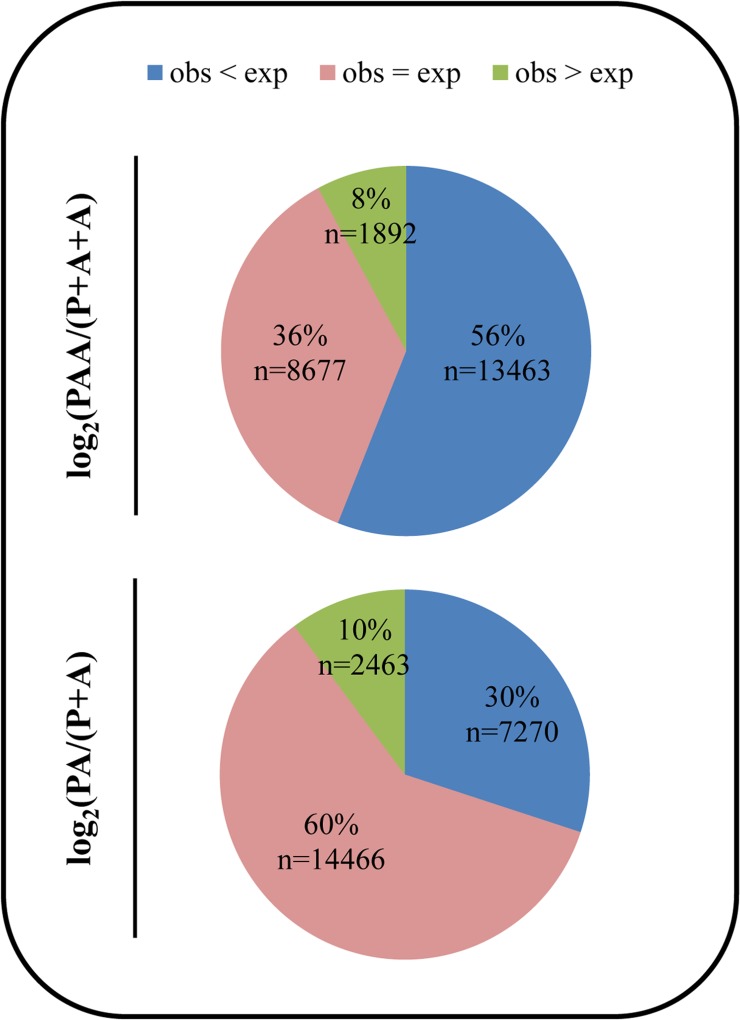
Gene expression additivity in hybrids. Additivity for each transcript was calculated by dividing the observed FPKM value by the expected FPKM value. The expected values were obtained using the expression values of the non-hybrid diploids—AA and PP. The expected value for PAA is (PP/2)+(AA/2)+(AA/2) and the expected value for PA is (PP/2)+(AA/2). Transcript were than evaluated as having lower observed expression then the additivity expectation (obs<exp), observed expression similar to the additivity expectation (obs = exp) or higher observed expression then the additivity expectation (obs>exp). The percentages of transcripts in each category, for both a) PA and b) PAA are represented.

We observed that for liv-PAA only 36% of transcripts are represented in the range of the expected/additive expression level, and from the transcripts that are not additively expressed, a very significant (χ-text, p<0.001) majority (56% of the total) are under-expressed compared to the additivity expectations (Fig. 4). Hence, in triploid hybrids gene expression in the liver is mostly negatively non-additive.

In the case of the diploid hybrid (PA), we observed that the percentage of additively expressed transcripts rises to more than half of the transcripts (60%) (Fig. 4). From the non-additively expressed ones a significant (χ-text, p<0.001) majority (30% of the total) is also under-expressed in comparison to the additivity expectations (Fig. 4).

### Similar gene expression and dosage compensation

We quantified the similarly expressed transcripts (SE) between each pair of 3n vs 2n genomotypes, both in juveniles and liver data sets (S6 Table). Focusing on the comparisons between hybrids (Table 1), we observe that 64% of the transcripts in juveniles and 44% in the livers are represented similarly in diploids and triploids. Within the SE group we also evaluated the occurrence and/or extension of strictly diploid expression levels in triploids (fold change equal to 1) and also of increased expression in triploids proportional to dosage increase (1.5 fold higher). So, we sorted the SE transcripts into 4 classes. Class I comprises the compensated transcripts, with ratio PAA/PA approximately equal to 1 and within the interval] 0.75;1.25[; in class II are the dosage sensitive ones, with ratio approximately equal to 1.5 and within] 1.25;1.75[; in class III are transcripts affected by some repression, with ratios lower than 0,75 and; in class IV are transcripts overexpressed, with ratios higher than 1.75 (Table 1). The results show that in triploid hybrids more than one third of the SE transcripts are strictly dosage compensated to the diploid hybrid level (45% in juveniles and 35% in liver) (Table 1). On the other hand, there is a much smaller representation of SE transcripts that follow the “1.5-fold rule”, being expressed proportionally to gene dosage (17% in juveniles and 13% in liver) (Table 1). Of notice is that in both data sets only a very small percentage of the SE PAA transcripts (4% in juveniles and 3% in livers) present an expression level higher than 1.5 fold (class IV), while 34% in juveniles and 49% in livers are repressed beyond dosage compensation (class III).

**Table 1:** Similarly expressed transcripts (SE) between triploid and diploid S. alburnoides hybrids in juveniles and livers.

Comparisons	SE (% of total)		SE *per* class	% of SE	% of total
**Juveniles *PAA/PA***	58076 **(64%)**	**I**	26376	45	**29**
		**II**	9935	17	**11**
		III	19672	34	22
		IV	2093	4	2
**Liver *PAA/PA***	10068 **(44%)**	**I**	3508	35	**15**
		**II**	1308	13	**6**
		III	4947	49	21
		IV	305	3	1

Total numbers and percentages of SE unigenes (juveniles) or mapped genes (livers) and total numbers and percentages of SE’s per expression class.

### Differential expression

Considering the significance criteria for differential gene expression (see material and methods) we quantified the significantly different expressed transcripts between diploid and triploid juveniles and livers (S7 Table). Focusing on the comparisons between hybrids (Table 2), we observed that 22.5% of unigenes in juveniles and 0.83% genes in livers are DE between diploids and triploids. Also, both for juveniles and livers the significant majority of the DE transcripts are higher represented in PAs then in PAAs, despite the higher gene dosage in triploids (Table 2).

**Table 2:** Differentially expressed transcripts (DE) between triploid and diploid S. alburnoides hybrids in juveniles and livers.

Comparisons	DE (% of total)	DE group	DE *per* group	% of DE	% of total
**Juveniles *PAA/PA***	20468 **(22.5%)**	DEH	6813	33	7,5
		DEL	13655	67	15
**Livers *PAA/PA***	195 **(0.83%)**	DEH	41	21	0,17
	DEL	154	79	0. 65

Total numbers and percentages of differently expressed (DE) unigenes in juveniles or mapped genes in livers. DE's were divided in two groups: significantly higher expressed in PAA compared to PA (DEH), and significantly lower expressed in PAA compared to PA (DEL).

### Functional enrichment analysis

We used the annotated *de novo* assembled transcriptome of juveniles and the mapping of the *S*. *alburnoides* liver transcriptome to the Zebrafish genome to perform a functional analysis [35]. In order to look for the biological context for the gene expression dosage regulation observed between diploid and triploid hybrids, we performed a GO and a KEGG pathway enrichment analysis in DE and SE groups.

We found significant functional enrichment in both DE and SE groups in juveniles (Table 3) and livers (Table 4), and the analysis is quit consistent between the two data sets. Briefly, the SE group is enriched in terms associated with metabolic processes, intracellular parts and constitutes of the ribosomes, while DE is mostly enriched in terms associated with the cell membrane (e.g. transport, adhesion, motility). From the KEGG pathway analysis in the SE group we observed that it is consistent with an enrichment of ribosomal components and ribosomal-linked pathways in both data sets. KEGG pathway analysis of the DE group of juveniles is significantly enriched in components of the circadian rhythm, Wnt signaling and melanogenesis pathways. The DE group in the liver data set is significantly enriched in components of the sphingolipid metabolism and PPAR signaling pathways. Within the SE group we also looked for differential functional enrichment between classes I and II in both juveniles and liver data sets (Table 5). Within our criteria of significance, no significant functional enrichment was detected in class I, both in juveniles and livers. Class II of juveniles is enriched in terms linked to ribosomal complex, to the respiratory chain and to the hemoglobin complex. Livers class II is enriched in terms linked to ribosomes.

**Table 3:** Functional enrichment in GO terms and KEGG pathways of PAA vs PA similarly expressed and differentially expressed gene groups for juveniles.

		Term	#	FE	p-val.
**SE**	BP	catabolic process	326	1,1	4,6E-3
	BP	macromolecule metabolic process	1993	1,0	3,6E-2
	BP	primary metabolic process	2496	1,0	4,7E-2
	CC	intracellular part	2829	1,0	6,7E-4
	BP	ribonucleoprotein complex	195	1,1	7,1E-4
	BP	cell part	4913	1,0	6,9E-4
	BP	intracellular	3450	1,0	6,0E-4
	BP	intracellular organelle	2358	1,0	6,3E-4
	MF	structural constituent of ribosome	94	1,1	3,0E-2
	KEGG	Spliceosome	93	1,2	1,4E-3
**DE**	BP	**cell adhesion**	85	1,7	2,1E-6
	BP	cellular developmental process	154	1,3	1,5E-2
	BP	**cellular component morphogenesis**	61	1,5	1,0E-2
	BP	anatomical structure development	282	1,2	8,5E-3
	BP	**cell motion**	56	1,5	4,8E-2
	BP	anatomical structure morphogenesis	169	1,2	5,0E-2
	CC	**extracellular region part**	57	1,5	1,2E-2
	CC	**extracellular matrix**	37	1,6	4,7E-2
	MF	signal transducer activity	272	1,3	3,9E-5
	MF	ion binding	620	1,1	1,2E-3
	KEGG	**Circadian rhythm**	12	3,1	3,2E-2
	KEGG	**Wnt signaling pathway**	43	1,5	4,7E-2
	KEGG	**Melanogenesis**	31	1,6	3,9E-2

(BP) Biological process, (MF) molecular function, (CC) cellular component, (KE) KEGG pathway, (#) number of transcripts, (FE) fold enrichment, (p) Benjamini corrected p-value. GO enrichment analysis was performed at the secondary classification of terms. Terms with FE ≥ 1.5 [35] are in bold.

**Table 4:** Functional enrichment in GO terms and KEGG pathways of PAA vs PA similarly expressed and differentially expressed gene groups for livers.

		Term	#	FE	p-val.
**SE**	BP	cellular metabolic process	1442	1,1	1,0E-16
	BP	macromolecule metabolic process	1259	1,2	5,3E-16
	BP	primary metabolic process	1529	1,1	5,9E-11
	BP	**ribonucleoprotein complex biogenesis**	42	1,8	1,8E-5
	BP	nitrogen compound metabolic process	663	1,1	2,0E-5
	BP	biosynthetic process	656	1,1	1,4E-4
	BP	catabolic process	205	1,2	9,0E-3
	BP	macromolecular complex subunit org.	82	1,3	9,4E-3
	BP	**establishment of RNA localization**	18	1,9	1,9E-2
	BP	**translational initiation**	24	1,7	2,0E-2
	CC	**ribonucleoprotein complex**	185	1,6	2,3E-18
	CC	intracellular	2021	1,1	5,3E-15
	CC	intracellular part	1652	1,1	4,5E-12
	CC	intracellular organelle	1369	1,1	4,7E-9
	CC	**organelle lumen**	138	1,5	1,3E-8
	CC	membrane-bounded organelle	1164	1,1	8,1E-8
	CC	cell part	2750	1,0	2,9E-4
	CC	intracellular organelle part	446	1,1	2,5E-3
	CC	organelle part	446	1,1	2,5E-3
	CC	non-membrane-bounded organelle	322	1,1	4,6E-3
	MF	nucleic acid binding	1002	1,2	1,2E-15
	MF	**structural constituent of ribosome**	104	1,7	9,3E-13
	MF	nucleotide binding	793	1,1	1,5E-7
	MF	**translation factor activity**	60	1,7	1,3E-6
	MF	nucleoside binding	501	1,1	1,6E-3
	MF	transferase activity	630	1,1	1,9E-3
	MF	ion binding	1052	1,1	4,2E-3
	MF	ligase activity	116	1,2	2,0E-2
	KEGG	**Ribosome**	79	2,0	2,1E-19
	KEGG	**Spliceosome**	89	1,7	6,5E-10
	KEGG	Ubiquitin mediated proteolysis	76	1,4	3,6E-3
	KEGG	**RNA degradation**	38	1,5	2,2E-3
**DE**	BP	**transport**	28	2,7	1,5E-5
	BP	**establishment of localization**	28	2,7	9,1E-6
	BP	**transmembrane transport**	11	3,6	1,1E-2
	CC	**membrane**	34	1,5	3,6E-2
	CC	**apical part of cell**	3	37,5	2,3E-2
	MF	**substrate-specific transporter activity**	17	4,3	1,7E-5
	MF	**hydrolase activity**	26	2,5	1,1E-4
	MF	**transmembrane transporter activity**	13	3,5	1,6E-3
	KEGG	**Sphingolipid metabolism**	4	16,8	2,2E-2
	KEGG	**PPAR signaling pathway**	4	13,3	2,2E-2

BP) Biological process, (MF) molecular function, (CC) cellular component, (KE) KEGG pathway, (#) number of transcripts, (FE) fold enrichment, (p) Benjamini corrected p-value. GO enrichment analysis was performed at the secondary classification of terms. Terms with FE ≥ 1.5 [35] are in bold.

**Table 5:** Differential functional enrichment in GO terms and KEGG pathways between class I and II, both in juvenile and liver data sets.

Juveniles					
		Term	#	FE	p-val.
**Class I**		No significant enrichment			
		**Term**	#	**FE**	**p-val.**
**Class II**	BP	heterocycle biosynthetic process	21	2,9	3,9E-3
	BP	tetrapyrrole metabolic process	12	4,0	1,2E-2
	BP	tetrapyrrole biosynthetic process	11	4,2	1,2E-2
	MF	structural molecule activity	65	1,6	3,4E-2
	MF	heme-copper terminal oxidase activity	10	4,1	3,7E-2
	MF	cytochrome-c oxidase activity	10	4,1	3,7E-2
	MF	oxidoreductase activity, acting on heme group	10	4,1	3,7E-2
	KEGG	Ribosome	21	2,6	5,7E-3
**Liver**					
		**Term**	#	**FE**	**p-val.**
**Class I**		No significant enrichment			
		**Term**	#	**FE**	**p-val.**
** Class II**	BP	translation	71	3,0	1,4E-16
	CC	ribosome	62	3,4	3,7E-20
	CC	ribonucleoprotein complex	65	2,3	1,6E-10
	CC	intracellular non-membrane-bounded organelle	82	1,7	6,6E-6
	CC	non-membrane-bounded organelle	82	1,7	6,6E-6
	CC	ribosomal subunit	11	3,8	6,3E-3
	CC	small ribosomal subunit	7	4,6	4,8E-2
	MF	structural constituent of ribosome	60	4,3	8,5E-24
	MF	structural molecule activity	72	3,4	2,9E-21
	KEGG	Ribosome	60	5,4	5,2E-35

(BP) Biological process, (MF) molecular function, (CC) cellular component, (KE) KEGG pathway, (#) number of transcripts, (FE) fold enrichment, (p) Benjamini corrected p-value. GO enrichment analysis was performed at the slim classification of terms.

### Relative transcriptome size

To estimate the relative size of the PAA transcriptome vs the PA transcriptome we used livers from both hybrid genomotypes and analyzed six target genes (*rpl8*, *rpl35*, *actb2*, *pabpc1a*, *eef1a* and *rpsa*) to obtain genome-normalized expression estimates through a qRT-PCR assay and transcriptome-normalized expression estimates from the RNA-Seq assay.

In order to estimate relative expression level per genome, we used a qRT-PCR strategy devised by [23] that normalizes cDNA amplification to genomic DNA (gDNA) amplification. The simultaneous RNA and gDNA extraction from the same cells preserves the in vivo RNA/gDNA ratios. This allowed us to normalize gene expression (cDNA amplification) to genome copy number (gDNA amplification), which directly gives the transcript abundance per genome. With this approach we quantified the expression per genome in the allotriploid (PAA) *S*. *alburnoides* relatively to its diploid counterpart (PA) for the six target genes (Table 6).

**Table 6:** Data and calculations used for estimating relative triploid vs diploid hybrid transcriptome size.

Gene	^1^Transcripts/genome (qRT-PCR; N = 5)		^2^Transcripts/cell (qRT-PCR; N = 5)	^3^Transcripts/transcriptome (RPKM; N = 1)	^4^Transcriptome size
	PAA/PA	SD	PAA/PA	PAA/PA	PAA/PA
***rpl8***	0,8	0,1	1,1	1,5	0,8
***eef1a***	0,8	0,1	1,1	1,3	0,9
***actb2***	0,6	0,2	0,9	0,6	1,6
***rpsa***	0,9	0,3	1,3	1,5	0,9
***pabpc1a***	0,8	0,2	1,2	1,0	1,2
***rpl35***	0,8	0,2	1,2	1,5	0,8
**media**	**0,8**		**1,2**	**1,0**	**1,0**
***SD***	0,1		0,1	0,3	0,3

^1^-Expression quantified by qRT-PCR, using total nucleic acid as the template for reverse transcription and normalization to genome copy number.

^2^- Because PAA has 3 genomes per cell, meaning 1,5x the amount of genomes per diploid cell, the transcripts/cell values for PAA/PA are equal to 1,5x the values for transcripts/genome.

^3^-For each target gene RPKM values were derived from the liver RNA-Seq data set.

^4^-Trascriptome size is determined by dividing “transcripts/cell” by “transcripts/transcriptome”.

Because PAA has three copies of each gene, for every two copies in PA diploids we calculated expression per cell in PAA relative to PA as 1,5x the relative expression per genome (Table 6).

**Fig 5:**
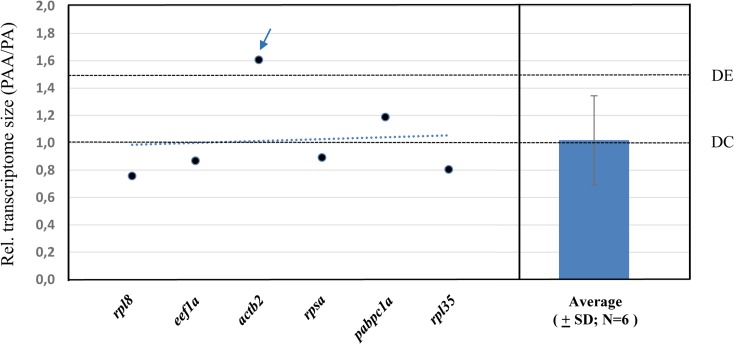
*S*. *alburnoides* PAA transcriptome size relative to the transcriptome of the diploid PA. Six individual gene-based estimates of relative transcriptome size and average estimate (±SD; N = 6) of the triploid hybrid transcriptome relative to the diploid hybrid transcriptome. DE represents the expected value if the PAA transcriptome experienced a genome-wide dosage effect. DC represents the expected value if PAA transcriptome experienced genome-wide dosage compensation. Dashed blue line represents the tendency curve of the sactterplot. The blue arrow indicates a possible outlier in the data set but that hypothesis was rejected (Tukey's Method).

The transcript abundance per transcriptome (RPKM values) for all six target genes were searched within the liver data set and the relative expression per transcriptome between liv-PAA and liv-PA was calculated (Table 6, Fig. 5). With this approach we obtained six independent estimates of the size of the triploid transcriptome relative to the diploid hybrid transcriptome. As expected, there was variation among individual gene estimates, but on average the PAA transcriptome was equal in size to the PA transcriptome. With these data we rejected the null hypothesis that the triploid hybrid transcriptome was subjected to a genome-wide dosage effect, as it was not increased 1.5 fold relative to the diploid hybrid transcriptome (P<0,0001; One sample *t*-test). On the other hand, the null hypothesis that PAA transcriptome was equal in size (genome wide dosage compensation) to the PA transcriptome was not rejected (P = 0.8867; One sample *t*-test).

## Discussion

### Transcriptome size and overall expression

To directly infer global expression dosage responses from the RNA-Seq transcript profiling experiments the transcriptomes compared must be of equal size [23]. When comparisons are made between different ploidy levels, intuitively this assumption is flawed due to the real genome-wide differences in gene dosage. However, in the *S*. *alburnoides* case, the hypothesis put forward in [12] and explored in the present work, is that there is a common “diploid” state of genic activity between diploid and triploid *S*. *alburnoides* individuals. Following the method described and implemented in [23], that couples transcript profiling data with a genome normalized qRT-PCR assay we estimated the liver transcriptome size of the *S*. *alburnoides* triploid hybrid (PAA) relatively to the liver transcriptome size of the diploid *S*. *alburnoides* hybrid (PA). We showed that the two compared transcriptomes are fairly the same size. This validates the direct use of the RNA-Seq transcript profiling experiments to infer the “gene by gene” global pattern of expression dosage responses between diploid and triploid *S*. *alburnoides*. Moreover, it supports at an overall scale the previous conjecture of transcriptional equivalence between diploid and triploid *S*. *alburnoides*.

### Allopolyploid genome regulation

One of the puzzling features of *S*. *alburnoides* complex is the extraordinary morphological similarity between PA and PAA individuals, which are even undistinguishable by morphometric characters [36]. Conversely, PP and AA genomotypes are easily distinguishable from each other and from the hybrids. The stable phenotypic similarity between PA and PAA hybrids could be interpreted as indication of similar gene expression between them [12, 37]. At its quantitative component, this hypothesis was corroborated on a small scale [12, 38], when diploid and triploid individuals were found to have similar expression levels for a small analyzed gene set. Conversely, our genome wide approach shows that, despite many genes do not present a significant differential expression between diploid and triploid hybrids, PAA gene expression levels are not globally identical to the ones of PA, or to any of the other diploids. So, the morphological similarity between PAA allotriploids and PA allodiploids is not due to strictly conserved mRNA levels between these genomotypes. On the other hand, the similarities may be at the relative transcriptional contribution of each genome type (P and A alleles) to the overall expression level of each gene, regardless of the total expression level of each gene on each hybrid. Other alternative to the above stated, would be the occurrence of conserved protein levels and identities between 2n and 3n hybrids [39]. So, the regulation between PA and PAA at the translational and post-translational levels and the allele specific contribution to the overall expression of each gene should be investigated in a near future.

However, at the transcriptional level, though we ruled out the hypotheses of a global strict full “functional diploidization” of triploids, the majority of transcripts is less represented in PAA then it is in PA and AA genomotypes. One could expect that the level of gene expression would change proportionally to the ploidy variation [40] and not the opposite. But, as an allopolyploid, *S*. *alburnoides* combines “ploidy rise” with hybridization and so, the effects associated to hybridization have to be considered. The pre-existing differences between the expression profiles of the parental genomotypes and also unpredictable effects of the complexities of an inter-genomic gene expression regulation are manifesting in the expression profiles of the *S*. *alburnoides* hybrids. We observed that the comparisons of both hybrids with PP are consistent with the comparative profile of AA vs PP, where the vast majority of transcripts are less represented in PP. Additionally, we observe a higher amount of transcripts that are less expressed in PAA then in PA. The observed “overcompensation” supports that between triploid and diploid hybrids, dosage compensation occurs, but it is not accurate. We can speculate that in PAA allotriploids the unbalanced genomic contribution and/or a faulty interaction between the different genomes might have to be compensated through allele specific expression regulation [12], and it may well be that the expression level of each allele might be quite variable and adaptable. So, as in plants and invertebrates, also in the *S*. *alburnoides* allopolyploid complex there is disruption, due to hybridization and anorthoploidy (odd ploidy) [41] of the quantitative assumptions of additivity, that are usually valid in the case of most homogenomic diploids and autopolyploids [9].

Another hypothesis we can put forward to explain the absence of a positive correlation between copy number and mRNA amount is that the expression level profiles of PAA and PA genomotypes, may be influenced by differences in cell size, which are expected to exist between individuals of different ploidy levels [42]. This hypothesis was not yet explored within the *S*. *alburnoides* complex, and is barely investigated in other organisms [43].

### Additivity

In any hybrid, gene expression is under the influence of divergent genomes, so new qualitative and quantitative gene expression networks are expectedly established, resulting from the interactions of the divergent alleles. In *S*. *alburnoides*, we observed that for most genes this expression level divergence from the parental genomotypes was not achieved by averaging the parental allelic contributions.

The analysis for additive expression showed that this occurs only for a subset of genes, both in diploid and triploid hybrids of *S*. *alburnoides* (Fig. 4). The occurrence of non-additive gene expression in hybrids and allopolyploids has been extensively reported in plants, for example in maize [44], rice [45] and *Arabidopsis* [46]. Also in animals as oysters [47] and *Drosophila* [13] the topic was explored and conclusions extended to the animal kingdom. However, for vertebrates, genome wide quantitative gene expression studies were missing.

Interestingly, the results of the available studies on plants and invertebrates are not all coincident. Several showed that the majority of genes are expressed additively [48, 49], while other studies found higher levels of nonadditive expression [13, 44]. The causes for these apparent discrepancies are not yet clear [50]. Anyway, the considerable body of data gathered so far shows a possible positive correlation between size of the fraction of the nonadditively expressed genes and the magnitude of heterotic response. Also, increasing the number of diverse genome copies in an allopolyploid, usually leads to increasingly greater magnitudes of heterosis [51]. Nevertheless, there is no consensus about the amount or identity of the nonadditively expressed genes [50, 52]. Concerning the *S*. *alburnoides* complex, the phenomenon of heterosis has been barely addressed, except for a few comparisons of growth and reproductive traits between diploid and triploid hybrids [53] and a comparative morphometric study [36], where the AA and PP parental genomotypes were not included. From these studies, mostly non-significant differences between diploid and triploid hybrids have been found, except for a marginal longevity increase in triploids. Yet, the PAA genomotype is far more frequent in the natural populations than PA. So, if we consider the number of non-additively expressed transcripts as an indicator of heterosis, PAA *S*. *alburnoides* are favored since the amount of additively expressed transcripts is higher than in PA genomotype. Also, according the Bateson-Dobzhansky-Muller Model a lower fitness in hybrids might result from a bad interaction between divergent genomes due to the differential capacity of interaction between their proteins [54]. In this light, allopolyploid individuals have better chances to evade this weakness. Allopolyploids have more options to non-additively combine allele-specific regulated expressions and so, have higher chances to achieve an optimized and more functional expression pattern than one achieved merely additively.

### Dosage responses across the *S*. *alburnoides* hybrid transcriptome—Expression level regulation

In our genome wide prospection for gene expression dosage compensation, a genome wide regulatory mechanism that brings all genic activity of triploids to the diploid state, in a “strict diploidization” of triploids was not seen. However, in both juveniles and liver data sets, we found a considerable fraction of all transcripts (29% in juveniles and 15% in livers) that really suffices the most stringent parameter definition for “fully dosage compensated”. So, “diploidization” might not mean that all genes are down regulated to the diploid level, but only those that need to be “diploidized” in RNA amount to function correctly. Also, a considerable part of all transcripts (around half) do not have a significantly different representation between diploid and triploid hybrids (SE group), and from the SE transcripts that do not belong to class I, the majority are lower represented in PAA than in PA (class III). In fact, there is only a very small percentage of triploid transcripts that are represented strictly proportionally to gene dosage (class II) or even higher (class IV). Thus, our results show a significant “diploidization” in triploid PAA genomotype, but not as a strictly regulated and fine-tuned phenomenon. That is consistent with a switch-like way to regulate the mRNA concentrations, where transcription is turned “on” or “off”, regardless of exact concentrations [55], but within boundaries of similar expression.

The mechanisms involved in this regulation of gene expression in *S*. *alburnoides* triploids vs diploids are still elusive, but miRNA’s were recently pointed [37] as significant regulators for the functional stability of triploidy in the *S*. *alburnoides* complex [37].

The quantitative PAA/PA gene expression analysis in juveniles and livers are conceptually coincidental so, most conclusions should be valid both at the single tissue gene expression regulation as at the full-body scale. There is also convergence between our study and the ones the ones that preceded it. For the same *S*. *alburnoides* genes analyzed in [12] and [38] we inspected the PAA/PA expression obtained in our study. All were place inside the SE group (data not shown), except *amh* and *dmrt1* (that are not expected to fit with the dataset).

### Functional context of the *S*. *alburnoides* genome regulation

In both, juveniles and liver data sets, the SE group is enriched in terms related to the basal biological maintenance of the cells (eg. metabolism), and mostly in ribosome-linked terms. But regardless of the statistical significance for the enrichment (corrected p-value < 0.05), the fold enrichment of each term is approximately 1. So, in the context of global expression, this enrichment may not be meaningful [35] or may indicate that between triploid and diploid hybrids genes with expression within boundaries of similar RNA amount can occur at any quantity without compromising there function. This expression level flexibility may be a strong contributor to overcome the allopolyploid “genomic shock”. Also it gives an immediate evolutionary advantage to the (allo)polyploids.

Within the SE group, we looked for functional enrichment in the strictly dosage compensated class (I) but no significant enrichment was found. That reinforces the previous idea, yet the very small class II, composed of genes with expression strictly proportional to gene dosage, presents a significant enrichment in terms associated to multi-subunit complexes, namely ribosomes. However, the detection of dosage sensitivity in genes whose products are part of multi-subunit complexes is in accordance with the gene balance hypothesis [40, 52], which posits that changes in the stoichiometry of the individual subunits would be deleterious.

The functional enrichment of the DE gene group may shed some light on phenotypic differences between PAA and PA genomotypes. We verified that in both, the liver and juvenile data sets the GO term enrichment is mostly associated to cell surface and to processes intimately linked to the cells membrane. Previously, it was described the same enrichment for differential expression between budding yeasts (*S*. *cerevisiae*) with different ploidy levels [42]. The authors suggested that the differential gene expression observed between ploidy levels was due to cell size and geometry differences between yeasts of different ploidies and not directly to the gene dosage increase [42]. In addition to yeasts, in many other polyploid organisms, including fish [56, 57, 58], the nucleus and cell volumes expand proportionally to accommodate the enlarged genome of polyploid cells [56]. However, there is a reduction in surface area relative to cell volume [43]. Consequently, the interactions between surface and cytoplasmic signaling, transport of metabolites and the cellular component organization are expected to be affected.

## Concluding Remarks

To our knowledge, this is the first study that globally quantitatively and comparatively profiled by mRNA-seq the transcriptomes of diploid and triploid forms of an allopolyploid vertebrate organism.

Our results point towards a certain level of flexibility of expression within a range of mRNA amounts per locus between diploid and triploid hybrids of the *S*. *alburnoides* complex. For these allotriploids, gene expression levels are similar to the ones of allodiploids but are neither genome wide strictly diploidized nor strictly proportional to gene dosage. The occurrence of a non-fine-tuned expression regulation at the transcription level might be a key factor for the evolutionary success of allopolyploids. Similar to nucleotide sequence variation, variability at the mRNA expression levels may be also a source for regulatory adaptation to selective pressures. Moreover, the evolution of new functions and subfunctionalizations from redundant genes, that are well known to occur in the allopolyploid situation, are probably facilitated in a context of expression dosage plasticity.

In conclusion, this work illustrates how a successful allopolyploid vertebrate transcriptionally deals with the genomic stress derived from hybridization and polyploidy and may shed some light on important features of genome evolution in allopolyploids.

## Supporting Information

S1 Table Output Statistics of sequencing for juveniles’ data set. (DOCX)

S2 Table Sequencing and mapping statistics for livers data set. (DOCX)

S3 Table Statistics of assembly quality for juveniles’ data set. (DOCX)

S4 Table Summary of annotation results for juveniles’ data set (cds information). (DOCX)

S5 Table qRT-PCR primers. (XLSX)

S6 Table Similarly expressed transcripts (SE) between each pair of 3n vs 2n *S*. *alburnoides* genomotypes, both in juveniles and liver data sets. (DOCX)

S7 Table Significantly differently expressed (DE) transcripts between each pair of 3n vs 2n S. alburnoides genomotype, both in juveniles and liver data sets. (DOCX)
