# Supplementary material for: Gene Expression Dosage Regulation in an Allopolyploid Fish
Source: PLoS One. 2015 Mar 19;10(3):e0116309. doi: 10.1371/journal.pone.0116309 (PMC4366067; doi:10.1371/journal.pone.0116309)
Supplement: S1 Table — (DOCX) [file pone.0116309.s001.docx]

| **Table S1:** Output Statistics of sequencing for juveniles’ data set | | | | | | |
| --- | --- | --- | --- | --- | --- | --- |
| **Samples** | **Total Raw Reads** | **Total Clean Reads** | **Total Clean Nucleotides (nt)** | **Q20 percentage** | **N %** | **GC %** |
| **juv_AA** | 86.210.418 | 80.706.646 | 7.263.598.140 | 98.18% | 0.00% | 49.49% |
| **juv_PA** | 87.256.908 | 82.315.202 | 7.408.368.180 | 98.18% | 0.01% | 49.68% |
| **juv_PAA** | 84.446.254 | 79.595.526 | 7.163.597.340 | 98.16% | 0.00% | 49.31% |
