# Supplementary material for: Gene Expression Dosage Regulation in an Allopolyploid Fish
Source: PLoS One. 2015 Mar 19;10(3):e0116309. doi: 10.1371/journal.pone.0116309 (PMC4366067; doi:10.1371/journal.pone.0116309)
Supplement: S2 Table — (DOCX) [file pone.0116309.s002.docx]

| **Table S2:** Sequencing and mapping statistics for livers data set | | | | | |
| --- | --- | --- | --- | --- | --- |
| **Code** | **Total reads*** | **Mapped reads** | **Properly paird (%)** | **Fragments** | **Singletons (%)** |
| **liv-AA** | 55691260 | 32784631 (59%) | 17240502 (31%) | 12521017 | 7742597 (13,9%) |
| **liv-PP** | 45463238 | 29025241 (64%) | 16141464 (35%) | 11234018 | 6557178 (14,5%) |
| **liv-PA** | 41955914 | 23366703 (56%) | 113258036 (27%) | 861058 | 6146187 (14,7%) |
| **liv-PAA** | 56266096 | 31553986 (56%) | 15357742 (27)%) | 11574116 | 8405754 (14,9) |
| * also QC-passed reads | | | | | |
