# Supplementary material for: Gene Expression Dosage Regulation in an Allopolyploid Fish
Source: PLoS One. 2015 Mar 19;10(3):e0116309. doi: 10.1371/journal.pone.0116309 (PMC4366067; doi:10.1371/journal.pone.0116309)
Supplement: S3 Table — (DOCX) [file pone.0116309.s003.docx]

| **Table S3:** Statistics of assembly quality for juveniles’ data set | | | | | | | | |
| --- | --- | --- | --- | --- | --- | --- | --- | --- |
| **Sample** |  | **Total Number** | **Total Length**  **(nt)** | **Mean Length**  **(nt)** | **N50** | **Total Consensus Sequences** | **Distinct Clusters** | **Distinct Singletons** |
| **juv_AA** | Contig | 141,478 | 64,387,301 | 455 | 1008 | - | - | - |
| **juv_PAA** |  | 176,458 | 61,061,715 | 346 | 641 | - | - | - |
| **juv_PA** |  | 165,484 | 64,691,831 | 391 | 825 | - | - | - |
| **juv_AA** | Unigene | 89,668 | 75,011,295 | 837 | 1543 | 89,668 | 19,292 | 70,376 |
| **juv_PAA** |  | 96,276 | 62,638,402 | 651 | 1079 | 96,276 | 24,034 | 72,242 |
| **juv_PA** |  | 94,919 | 71,915,349 | 758 | 1366 | 94,919 | 26,222 | 68,697 |
| **All*** |  | 92,137 | 91,274,231 | 991 | 1731 | 92,137 | 36,870 | 55,267 |
| * The contigs from the 3 juveniles libraries | | | | | | | | |
