# Supplementary material for: Gene Expression Dosage Regulation in an Allopolyploid Fish
Source: PLoS One. 2015 Mar 19;10(3):e0116309. doi: 10.1371/journal.pone.0116309 (PMC4366067; doi:10.1371/journal.pone.0116309)
Supplement: S4 Table — (DOCX) [file pone.0116309.s004.docx]

| **Table S4:** Summary of annotation results for juveniles’ data set (cds information) | | | | | |
| --- | --- | --- | --- | --- | --- |
| **Sequence File** | **NR** | **SwissProt** | **KEGG** | **COG** | **ALL** |
| **juv_AA** | 51,173 | 44,715 | 36,636 | 14,849 | 71,979 |
| **juv_PA** | 53,067 | 45,830 | 37,219 | 14,664 | 75,095 |
| **juv_PAA** | 54,769 | 47,333 | 37,663 | 13,871 | 76,641 |
| **All*** | 52,460 | 46,149 | 38,361 | 16,967 | 74,463 |
| *joining the 3 juveniles libraries | | | | | |
