# Supplementary material for: Gene Expression Dosage Regulation in an Allopolyploid Fish
Source: PLoS One. 2015 Mar 19;10(3):e0116309. doi: 10.1371/journal.pone.0116309 (PMC4366067; doi:10.1371/journal.pone.0116309)
Supplement: S6 Table — (DOCX) [file pone.0116309.s006.docx]

|  | | | | | | | | | | | | |
| --- | --- | --- | --- | --- | --- | --- | --- | --- | --- | --- | --- | --- |
| **Table S6:** Similarly expressed transcripts (SE) between each pair of 3n vs 2n *S. alburnoides* genomotypes, both in juveniles and liver data sets. | | | | | | | | | | | | |
|  |  | **Comparisons** |  | **SE** |  | **SE class** |  | **SE *per* class** |  | **% of SE** |  | **% of total** |
|  |  |  |  |  |  |  |  |  |  |  |  |  |
| **juveniles** |  | ***PAA/PA*** |  | 58076 **(64%)** |  | **I** |  | 26376 |  | 45% |  | 29% |
|  |  |  |  |  |  | **II** |  | 9935 |  | 17% |  | 11% |
|  |  |  |  |  |  | III |  | 19672 |  | 34% |  | 22% |
|  |  |  |  |  |  | IV |  | 2093 |  | 4% |  | 2% |
|  |  |  |  |  |  |  |  |  |  |  |  |  |
|  |  | ***PAA/AA*** |  | 49778 **(55%)** |  | **I** |  | 20925 |  | 42% |  | 23% |
|  |  |  |  |  |  | **II** |  | 7148 |  | 14% |  | 8% |
|  |  |  |  |  |  | III |  | 20087 |  | 40% |  | 22% |
|  |  |  |  |  |  | IV |  | 1618 |  | 3% |  | 2% |
|  |  |  |  |  |  |  |  |  |  |  |  |  |
| **livers** |  | ***PAA/PA*** |  | 10068 **(44%)** |  | **I** |  | 3508 |  | 35% |  | 15% |
|  |  |  |  |  |  | **II** |  | 1308 |  | 13% |  | 6% |
|  |  |  |  |  |  | III |  | 4947 |  | 49% |  | 21% |
|  |  |  |  |  |  | IV |  | 305 |  | 3% |  | 1% |
|  |  |  |  |  |  |  |  |  |  |  |  |  |
|  |  | ***PAA/AA*** |  | 9075 **(38%)** |  | **I** |  | 3823 |  | 42% |  | 16% |
|  |  |  |  |  |  | **II** |  | 1359 |  | 15% |  | 6% |
|  |  |  |  |  |  | III |  | 3553 |  | 39% |  | 15% |
|  |  |  |  |  |  | IV |  | 340 |  | 4% |  | 1% |
|  |  |  |  |  |  |  |  |  |  |  |  |  |
|  |  | ***PAA/PP*** |  | 9013 **(41%)** |  | **I** |  | 3473 |  | 39% |  | 16% |
|  |  |  |  |  |  | **II** |  | 2970 |  | 33% |  | 13% |
|  |  |  |  |  |  | III |  | 990 |  | 11% |  | 4% |
|  |  |  |  |  |  | IV |  | 1580 |  | 17% |  | 7% |
|  | | | | | | | | | | | | |
| Total numbers and percentages of SE unigenes (juveniles) and mapped genes (livers) and total numbers and percentages of SE’s *per* expression class. | | | | | | | | | | | | |
