# Supplementary material for: Gene Expression Dosage Regulation in an Allopolyploid Fish
Source: PLoS One. 2015 Mar 19;10(3):e0116309. doi: 10.1371/journal.pone.0116309 (PMC4366067; doi:10.1371/journal.pone.0116309)
Supplement: S7 Table — (DOCX) [file pone.0116309.s007.docx]

| **Table S7:** Significantly differently expressed (DE) transcripts between each pair of 3n vs 2n S. alburnoides genomotype, both in juveniles and liver data sets | | | | | | | | |
| --- | --- | --- | --- | --- | --- | --- | --- | --- |
|  |  | **Comparisons** |  | **DE** | | | | |
|  |  |  |  |  |  |  |  |  |
| **juveniles** |  | ***PAA/PA*** |  | 20468 **(22.5%)** |  | Up |  | 6813 **(7,5%)** |
|  |  |  |  |  |  |  |  |  |
|  |  |  |  |  |  |  |  |  |
|  |  |  |  |  |  | Down |  | 13655 **(15%)** |
|  |  |  |  |  |  |  |  |  |
|  |  |  |  |  |  |  |  |  |
|  |  | ***PAA/AA*** |  | 30024 **(33%)** |  | Up |  | 12504 **(14%)** |
|  |  |  |  |  |  |  |  |  |
|  |  |  |  |  |  |  |  |  |
|  |  |  |  |  |  | Down |  | 17520 **(19%)** |
|  |  |  |  |  |  |  |  |  |
|  |  |  |  |  |  |  |  |  |
| **liver** |  | ***PAA/PA*** |  | 195 **(0.83%)** |  | Up |  | 41 **(0.17%)** |
|  |  |  |  |  |  |  |  |  |
|  |  |  |  |  |  |  |  |  |
|  |  |  |  |  |  | Down |  | 154 **(0.65%)** |
|  |  |  |  |  |  |  |  |  |
|  |  |  |  |  |  |  |  |  |
|  |  | ***PAA/AA*** |  | 261 **(1.1%)** |  | Up |  | 64 **(0.27%)** |
|  |  |  |  |  |  |  |  |  |
|  |  |  |  |  |  |  |  |  |
|  |  |  |  |  |  | Down |  | 197 **(0.83%)** |
|  |  |  |  |  |  |  |  |  |
|  |  |  |  |  |  |  |  |  |
|  |  | ***PAA/PP*** |  | 52 **(0.23%)** |  | Up |  | 39 **(0.18%)** |
|  |  |  |  |  |  |  |  |  |
|  |  |  |  |  |  |  |  |  |
|  |  |  |  |  |  | Down |  | 13 **(0.06%)** |
|  |  |  |  |  |  |  |  |  |
|  |  |  |  |  |  |  |  |  |
| Total numbers and percentages of differently expressed (DE) unigenes in juveniles and mapped genes in livers. DE's were divided in two groups: significantly higher expressed in PAA compared to PA (DEH), and significantly lower expressed in PAA compared to PA (DEL). | | | | | | | | |
